# Improved Survival with Improved NAPRC Compliance: A Single-Institution Experience

**DOI:** 10.3390/jcm14196872

**Published:** 2025-09-28

**Authors:** Harry Wasvary, Jacob A. Applegarth, Scarlett Hao, Tyler A. Kowalczyk, Gayaneh Nazarian, Claire Bova

**Affiliations:** 1 Corewell Health William Beaumont University Hospital, Royal Oak, MI 48073, USA; 2 Oakland University William Beaumont School of Medicine, Rochester, MI 48309, USA; 3 Central Michigan University College of Medicine, Saginaw, MI 48602, USA

**Keywords:** rectal cancer, survival analysis, Nation Accreditation Program for Rectal Cancer (NAPRC)

## Abstract

**Background:** The National Accreditation Program for Rectal Cancer (NAPRC) was developed in 2017. This study investigates three-year survival after diagnosis of rectal cancer as a function of compliance with NAPRC standards. **Methods:** A prospective database recorded compliance with 15 NAPRC standards for patients diagnosed August 2019 through August 2021. This database was retrospectively reviewed for compliance and three-year survival after diagnosis. **Results:** Three groups were identified (low, moderate, and high compliance) without significant difference in age (*p* = 0.662), sex (*p* = 0.919), race (*p* = 0.88), or disease stage (*p* = 0.166) between groups. Compared to the least compliant group, both moderate- and high-compliance groups had statistically significant lower hazard ratios (HR 0.22 and HR 0.12, respectively). **Conclusions:** Increased compliance led to a significant survival benefit. Rectal cancer patients who received care adherent to at least eight components of the NAPRC standards had a significant survival benefit three years after diagnosis compared to patients with less compliance.

## 1. Introduction

Colorectal cancer is the second leading cause of cancer-related deaths in the United States [1]. Although the widespread use of screening colonoscopies has resulted in a decrease in overall colorectal cancer mortality, the outcomes for rectal cancer have historically been inconsistent [2]. Over the past twenty years, the effectiveness of treatments for rectal cancer in the U.S. has varied significantly. These differences are often associated with the expertise level of hospital staff, healthcare professionals’ competency, and the volume of cases managed [3].

Early attempts at standardization of rectal cancer care are reflected by the Consortium for Optimizing the Surgical Treatment of Rectal Cancer (OSTRiCH). Established in 2011, this consortium sought to address variability in rectal cancer care, such as documented disparities in length of stay [4], permanent stoma rates [5], and five-year mortality for high- versus low-volume surgeons [6], by designating Centers of Excellence (CoE) in rectal cancer care [7]. To be designated a CoE, an institution should adhere to five principles, including total mesorectal excision, specialized pathology, routine use of MRI, delivery of adjuvant and neoadjuvant therapy, and a multidisciplinary approach to rectal cancer care. Six years later, the results from one analysis found that of the then 350 OSTRiCH member institutions, the mean number of standards in compliance was 10.6 out of 22 for centers that responded, with only four centers projected to meet all standards [8].

To further address these disparities, the American College of Surgeons (ACS) established the National Accreditation Program for Rectal Cancer (NAPRC) in 2017 [9]. To achieve NAPRC accreditation, institutions are required to follow program guidelines and adhere to a strict set of patient care standards [9]. These standards follow fundamental principles of rectal cancer care and include reviewing pathology, staging with computed tomography (CT) and magnetic resonance imaging (MRI), and obtaining a carcinoembryonic antigen (CEA) level [10].

Findings from OSTRiCH centers demonstrated that institutions were ill-prepared to implement the strict standards developed by the NAPRC. However, previous work at our institution demonstrated that with diligent departmental guidance, acceptable levels of compliance can be achieved within 1 to 2 years of accreditation [11]. A previous retrospective review of our rectal cancer care examined rectal cancer patients diagnosed between 2016 and 2022 to compare compliance with guidelines before and after the hospital began NAPRC accreditation. The group of patients treated after the adoption of NAPRC standards demonstrated significantly improved compliance with pretreatment protocols, including obtaining MRI scans (*p* = 0.012), CT scans (*p* < 0.001), and measuring carcinoembryonic antigen (CEA) levels (*p* < 0.001) [11]. Additionally, there were notable improvements in postoperative measures, such as photographing surgical specimens (*p* < 0.001). Through diligent multidisciplinary team action, we were able to significantly improve our compliance with patient care standards.

Our institution began its journey toward NAPRC accreditation in August 2019 and received full accreditation in October of 2022. Beginning in 2019, with the NAPRC accreditation process, a prospective database was created to track patient compliance and outcomes at our institution. After demonstrating improved compliance when achieving full accreditation, we sought to examine the impact of increasing compliance with NAPRC standards on three year overall patient survival.

Objective:

This study aims to evaluate patients within a prospective database and investigate three-year overall survival outcomes of rectal cancer patients relative to compliance with NAPRC standards.

## 2. Materials and Methods

**Figure 1:**
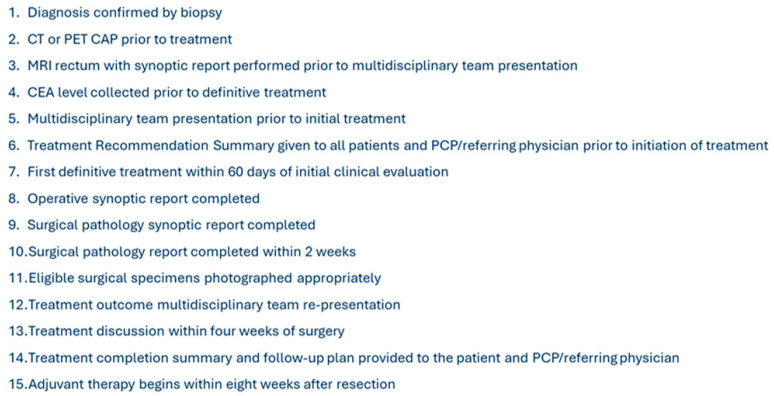
List of included accreditation standards.

A prospective database was used to document compliance with 15 NAPRC measures (Figure 1) for all rectal cancer patients treated at our institution from August of 2019 through August of 2021. Patients were considered compliant if they completed the measure as listed according to NAPRC standards. For example, if the patient underwent MRI with an appropriate synoptic report dictated by the radiology department prior to our multidisciplinary tumor board presentation, then they would be compliant with measure 3 (Figure 1). This database was then retrospectively reviewed for individual patient compliance with components of the NAPRC patient care standards. After total compliance with each of the 15 components had been documented, retrospective chart review was performed to assess for survival three years after diagnosis. Patients who elected for palliative measures or proceeded with a watch-and-wait approach, rather than oncologic resection, were excluded.

Demographic data including sex, race, and stage of disease at presentation were analyzed via Fisher’s exact test. Continuous demographic variables were assessed by a Kruskal–Wallis test, a non-parametric method used to test for a difference across the three levels of total compliance.

Eligible patients were analyzed for survival three years after diagnosis according to three comparison groups: compliance with 7 or fewer components, compliance with 8–12 components, and compliance with greater than 12 components. This analysis was performed via a log rank test with the creation of Kaplan–Meier survival curves.

Components of NAPRC care included in the study were further analyzed by multivariate regression to assess their individual contribution to overall survival benefit. Nine of fifteen standards had sufficient patients in each arm (compliant vs. non-compliant) to allow for statistical comparison using multivariate regression.

## 3. Results

**Figure 2:**
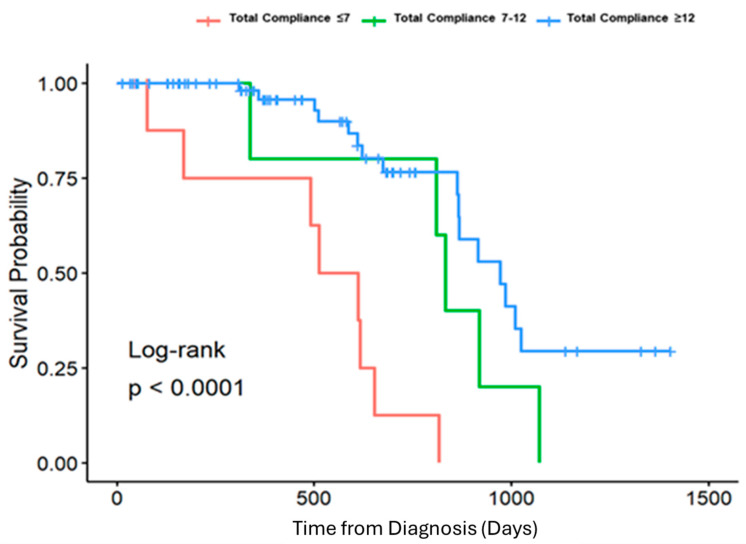
Kaplan-Meier survival curve with respect to total compliance among three groups (*p* < 0.0001).

**Figure 3:**
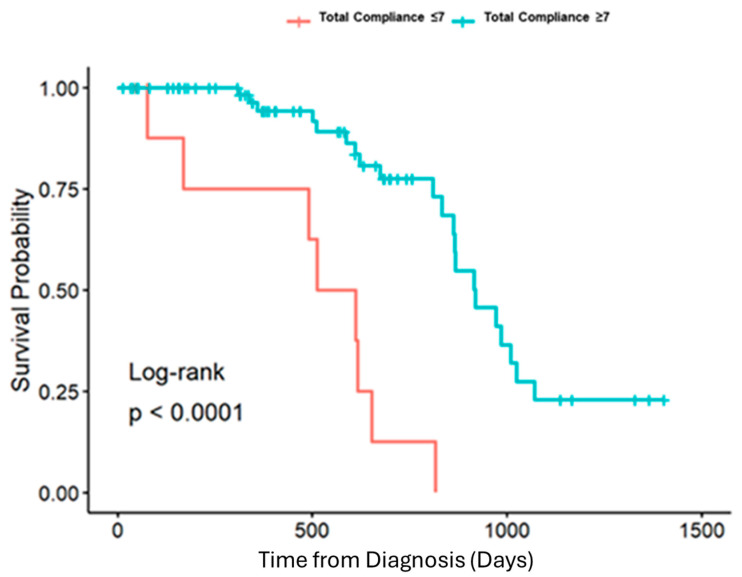
Kaplan-Meier survival curve with respect to total compliance with high versus low compliance (*p* < 0.0001).

A total of 87 patients met the inclusion criteria. A histogram of compliance data demonstrated three groups of patients: compliance with 7 or fewer components, compliance with 8–12 components, and compliance with greater than 12 components. There was no significant difference in age (*p* = 0.662), sex (*p* = 0.919), race (*p* = 0.88), or disease stage (*p* = 0.166) between the three comparison groups (Table 1). The survival rate at 3 years after diagnosis for each of these groups was as follows: compliance with 7 or fewer components was associated with a survival rate of 20%, compliance with 8–12 components was linked to a survival rate of 28.6%, and compliance with greater than 12 components resulted in a survival rate of 77.1%. A Log-rank test with Kaplan–Meier curves demonstrated a statistically significant difference in survival at three years between the comparison groups (Figure 2). Compared to the least compliant group, both moderate- and high-compliance groups had statistically significant lower hazard ratios (HR 0.22 [0.065–0.77] and HR 0.12 [0.044–0.31], respectively). However, compliance with 8–12 components and compliance with greater than 12 components did not significantly differ from each other. In sum, compliance with more than eight components led to a statistically significant survival benefit at three years in patients treated at our institution (Figure 3). Importantly, total compliance was analyzed as a continuous covariate, and for every additional measure completed, the probability of death at 3 years was decreased by 0.76 (HR 0.76 [0.67–0.87]).

**Figure 4:**
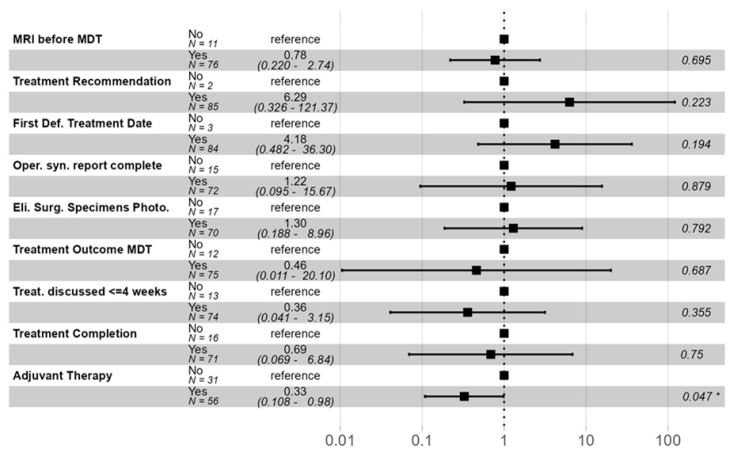
Multivariate Analysis of NAPRC Components. Multivariate analysis with hazard ratio for risk of death at three years after diagnosis. Only adjuvant therapy within 8 weeks of resection achieved statistical significance (*p* < 0.05). Significance with *p* < 0.05 denoted with (*).

When statistically possible given overall compliance and ability for comparison, components of NAPRC care included in our study were analyzed by multivariate regression to assess for contribution to this survival benefit (Figure 4). Only adjuvant therapy within eight weeks of resection was found to independently have a statistically significant effect on survival (*p* < 0.05).

## 4. Discussion

As an increasing number of institutions commit resources to become NAPRC accredited, it is important to monitor, evaluate, and report outcomes related to these efforts. Other studies have attempted to demonstrate improved outcomes with NAPRC accreditation but have demonstrated mixed findings.

Aggarwal et al. [12] also investigated short-term outcomes for two groups, pre-NAPRC accreditation and post-NAPRC accreditation, at their institution. While post-NAPRC patients were more likely to have complete pre-staging workup, tumor board presentations, CEA levels, and initiation of treatment within 60 days of diagnosis, there was no significant difference in short-term outcomes between the groups [12].

Brady et al. [13] investigated whether adherence to six out of thirteen NAPRC process measures correlated with improved survival. Process measures that were included in their analysis were captured in the National Cancer Database (NCDB) and included clinical staging, serum carcinoembryonic antigen level measured prior to starting definitive treatment, treatment started within 60 days of diagnosis, tumor regression grading performed, circumferential radial margin positivity assessed, and proximal and distal margin assessment on final pathology report. Their analysis revealed that compliance with these six measures was correlated with mortality benefit [13]. This significant result held true despite low overall compliance with included NAPRC measures. Researchers hypothesized that improving compliance would lead to improved survival rates for rectal cancer.

Harbaugh et al. [14] investigated the impact of NAPRC accreditation on outcomes after rectal cancer surgery. This study retrospectively analyzed Medicare beneficiaries aged 65 to 99 years with rectal cancer who underwent proctectomy from 2017 to 2020. Specifically, we examined the impact of NAPRC accreditation on primary outcomes following rectal cancer surgery, including differences in mortality (in-hospital, 30-day, and 1-year) as well as 30-day complications, readmissions, and reoperations. The study analyzed 1985 hospitals, 65 of which were NAPRC accredited [14]. The results indicated that hospitals with NAPRC-accreditation have lower risk-adjusted morbidity and mortality after a major rectal cancer surgery within one year compared to non-NAPRC hospitals. In particular, the in-hospital mortality was 1.1% vs. 1.3% (*p* = 0.002), 30-day mortality was 2.1% vs. 2.9% (*p* < 0.001), 30-day complications were 18.3% vs. 19.4% (*p* = 0.01), and 1-year mortality was 11.0% vs. 12.1% (*p* < 0.001) [14].

Similarly to Harbaugh et al [14]., we present our single-institution experience with NAPRC accreditation and its impact on mortality benefit. However, our study period was expanded to assess survival at three years after diagnosis, contributing to the available literature of long-term outcomes for NAPRC centers. When treated at our institution, increased compliance led to increased three-year overall survival. Rectal cancer patients who received care adherent to at least eight components of the NAPRC patient care standards had a significant survival benefit at three years compared to patients with less compliance. While timely adjuvant therapy was shown to be associated with survival benefit, this pattern may reflect a patient’s willingness to comply and follow up with providers. Additionally, as treatment patterns for rectal cancer have changed to include more neoadjuvant therapy, fewer rectal cancer patients will now require adjuvant therapy. Given this, we believe that the collaboration brought forth from NAPRC accreditation is the more likely driver of increased survival in the most compliant patients rather than any one single variable. Further work is needed to continue to monitor long-term survival and other oncologic outcomes. Future work will need to consider the outcomes of patients who elect to receive watch-and-wait care and how their outcomes will impact survival benefit as a function of NAPRC compliance.

## Figures and Tables

**Table 1:** Patient characteristics by total compliance. Patient demographics separated by compliance groups. No statistically significant difference was found between groups in regards to Age, Sex, Race, or stage of disease at time of diagnosis.

	Total Compliance <= 7		Total Compliance 8–12		Total Compliance > 12		Overall		*p*-Value
	Death (N = 8)	Survival (N = 2)	Death (N = 5)	Survival (N = 2)	Death (N = 16)	Survival (N = 54)	Death (N = 29)	Survival (N = 58)	
**Age at presentation**									
Mean (SD)	62.9 (13.9)	61.0 (7.07)	68.6 (16.9)	59.0 (12.7)	64.3 (12.9)	60.6 (11.2)	64.6 (13.5)	60.6 (11.0)	0.662
Median [Min, Max]	63.5 [41.0, 86.0]	61.0 [56.0, 66.0]	78.0 [43.0, 84.0]	59.0 [50.0, 68.0]	63.0 [44.0, 84.0]	62.0 [36.0, 92.0]	64.0 [41.0, 86.0]	62.0 [36.0, 92.0]	
**Sex**									
Female	5 (62.5%)	0 (0%)	2 (40.0%)	1 (50.0%)	8 (50.0%)	21 (38.9%)	15 (51.7%)	22 (37.9%)	0.919
Male	3 (37.5%)	2 (100%)	3 (60.0%)	1 (50.0%)	8 (50.0%)	33 (61.1%)	14 (48.3%)	36 (62.1%)	
**Race**									
White or Caucasian	6 (75.0%)	2 (100%)	4 (80.0%)	2 (100%)	12 (75.0%)	46 (85.2%)	22 (75.9%)	50 (86.2%)	0.88
Asian	0 (0%)	0 (0%)	0 (0%)	0 (0%)	0 (0%)	2 (3.7%)	0 (0%)	2 (3.4%)	
Black or African American	2 (25.0%)	0 (0%)	1 (20.0%)	0 (0%)	3 (18.8%)	5 (9.3%)	6 (20.7%)	5 (8.6%)	
Other	0 (0%)	0 (0%)	0 (0%)	0 (0%)	1 (6.3%)	1 (1.9%)	1 (3.4%)	1 (1.7%)	
**Stage**									
1	0 (0%)	0 (0%)	0 (0%)	1 (50.0%)	1 (6.3%)	6 (11.1%)	1 (3.4%)	7 (12.1%)	0.166
2	2 (25.0%)	0 (0%)	2 (40.0%)	1 (50.0%)	4 (25.0%)	15 (27.8%)	8 (27.6%)	16 (27.6%)	
3	2 (25.0%)	2 (100%)	1 (20.0%)	0 (0%)	7 (43.8%)	28 (51.9%)	10 (34.5%)	30 (51.7%)	
4	4 (50.0%)	0 (0%)	2 (40.0%)	0 (0%)	4 (25.0%)	5 (9.3%)	10 (34.5%)	5 (8.6%)	

## Data Availability

The original contributions presented in this study are included in the article. Further inquiries can be directed to the corresponding author.
